# Prognostic impact of dose reduced S-1 adjuvant chemotherapy in patients with pancreatic ductal adenocarcinoma: a retrospective multicenter study

**DOI:** 10.1007/s10147-025-02742-0

**Published:** 2025-04-26

**Authors:** Kazuki Kobayashi, Takahiro Einama, Yoichi Miyata, Asuma Ide, Naoto Yonamine, Takazumi Tsunenari, Mikiya Takao, Masato Yamadera, Makoto Nishikawa, Akifumi Kimura, Eiji Shinto, Hideki Ueno, Yoshifumi Beck, Yoji Kishi

**Affiliations:** 1 Department of Surgery, National Defense Medical College, 3-2 Namiki, Tokorozawa, Saitama 359-8513 Japan; 2 Department of Hepato-Biliary-Pancreatic Surgery, Center Hospital of the National Center for Global Health and Medicine, 1-21-1 Toyama, Shinjuku, Tokyo 162-8655 Japan; 3 Department of Surgery, Self-Defense Force Central Hospital, 1-2-24 Ikejiri, Setagaya, Tokyo 154-8532 Japan; 4 Department of Hepato-Biliary-Pancreatic Surgery and Pediatric Surgery, Saitama Medical Center, Saitama Medical University, 1981 Kamoda, Kawagoeshi, Saitama 350-8550 Japan

**Keywords:** Pancreatic ductal adenocarcinoma, S-1, Adjuvant chemotherapy, Total dose intensity, Dose reduced adjuvant chemotherapy

## Abstract

**Background:**

The standard adjuvant chemotherapy for pancreatic ductal adenocarcinoma (PDAC) in Japan is S-1; however, the impact of dose reduction on prognosis remains unclear. We have reported that total dose intensity (TDI) ≥ 62.5% indicates good prognosis. This multicenter retrospective study evaluated the prognostic impact of TDI ≥ 62.5% and reduced dosing in patients who underwent radical resection for PDAC across three institutions.

**Method:**

Patients were grouped into high-TDI (≥ 62.5%) and low-TDI (< 62.5%) based on this cutoff. We performed an inverse probability of treatment weighting (IPTW)-adjusted analysis and calculated relapse-free survival (RFS) and overall survival (OS). OS was also calculated for high TDI with TDI < 100% and TDI = 100%.

**Result:**

Among 487 patients, 274 were included: 152 in the low-TDI and 122 in high-TDI groups. Patient background was adjusted using IPTW based on factors that might influence TDI. The median RFS for low- and high-TDI was 8 and 18 months, respectively (p = 0.004). The median OS of low- and high-TDI groups was 20 and 50 months, respectively (p < 0.001). Among patients with high TDI, OS did not differ between those with TDI < 100% and those with TDI = 100% (median, 47 vs. 72 months, p = 0.208).

**Conclusion:**

It has been suggested that a partial dose reduction of S-1 as adjuvant chemotherapy for PDAC does not significantly impact prognosis.

## Introduction

Pancreatic cancer is associated with poor prognosis. Conversely, advancements in assessing resectability through preoperative imaging [1], the shift to less invasive surgical procedures such as laparoscopic and robotic surgery [2], and consideration of preoperative chemotherapy [3] have contributed to improved outcomes. Preoperative chemotherapy, including neoadjuvant chemotherapy (NAC) and adjuvant chemotherapy (AC), has substantially improved postoperative outcomes in patients with resectable tumors [4, 5], and perioperative chemotherapy has become an important factor in pancreatic cancer treatment alongside surgical interventions.

According to the findings of JASPAC-01 [6], the standard AC regimen in Japan involves the administration of S-1 for 6 months. However, approximately 40% of the patients in this study experienced dose reduction or suspension of S-1 due to adverse events, although the impact of these events on patient prognosis remains unclear.

We previously examined the prognostic impact of S-1 dose reduction using the total dose intensity (TDI) [7]. TDI was calculated using the following formula:

TDI = (real dose × actual administration days) / (ideal dose × ideal administration days) × 100 (%).

The TDI represents the total dose of chemotherapy and is known for its ease of calculation, as it does not account for the time factor. Our previous results suggested that a TDI of 62.5% was a feasible cutoff to obtain acceptable relapse-free survival (RFS) and overall survival (OS) improvements [7]. However, several limitations were present in that study. First, it was conducted at a single institution and included only a limited number of patients. Second, we did not compare the prognosis between the patients with TDI of ≥ 62.5% and < 100% and those with TDI = 100%. Third, a substantial imbalance in patient characteristics among the groups might have affected RFS and OS.

Therefore, in the present study, we conducted a multicenter retrospective study to evaluate the prognostic impact of a TDI of 62.5% after adjusting for patient backgrounds using inverse probability of treatment weighting (IPTW). In addition, we examined the prognostic impact of a reduced chemotherapy dose (TDI < 100%).

## Materials and methods

### Ethics statement

This study was approved by the Institutional Review Board of National Defense Medical College (Approval No. 4511). All participants provided informed consent. The study complied with the principles of the Declaration of Helsinki. All methods were performed in accordance with the relevant guidelines and regulations.

### Patients

We conducted a retrospective study involving three medical institutions in Japan: the National Defense Medical College Hospital, Self-Defense Force Central Hospital, and Saitama Medical Center. We selected patients who underwent surgical resection for pancreatic cancer between November 2011 and March 2019.

The inclusion criteria were as follows: (1) patients diagnosed with pancreatic cancer who underwent radical resection, including regional lymph nodes, and (2) availability of complete clinical data.

The exclusion criteria were as follows: (1) patients initially diagnosed with unresectable pancreatic cancer according to the National Comprehensive Cancer Network (NCCN) Guidelines [8]; (2) cases with a pathological diagnosis other than invasive ductal adenocarcinoma; (3) patients who underwent macroscopically noncurative (R2) resection; (4) patients who received AC with a regimen other than S-1; (5) Patients with TDI = 0% due to tumor recurrence or death; (6) patients with incomplete clinical data.

### Study design

We calculated the TDI for all patients and classified them into low (< 62.5%) and high (≥ 62.5%) TDI groups, using 62.5% as the cutoff value based on our previous report [7]. Moreover, patients with high TDI were further classified into three groups: over TDI (o-TDI, TDI > 100%), complete TDI (c-TDI, TDI = 100%), and reduced high TDI (rh-TDI, TDI ≥ 62.5% and < 100%). Patients with low TDI were further classified into two groups: reduced low TDI (rl-TDI, TDI > 0% and < 62.5%) and no TDI (n-TDI, TDI = 0%). Postoperative RFS and OS were compared between the high- and low-TDI groups. Postoperative OS was also compared the following groups: n-TDI vs rl-TDI, c-TDI vs rh-TDI, and o-TDI vs rh-TDI. Univariate and multivariate analyses were conducted to identify predictors of poor OS. Factors associated with low TDI were also evaluated.

### Data collection and definitions

Clinical data, treatment details, pathological characteristics, and survival outcomes were collected. Serum CA19-9 levels were measured after biliary drainage in patients with obstructive jaundice. Postoperative CA19-9 levels were defined as those measured at the first outpatient visit within 1–2 months after discharge. In the patients that AC was administered, the CA19-9 level just before the initiation of AC was used. The cutoff value for CA19-9 was set at 120 U/mL based on a previous study [9]. Surgical methods were determined based on the tumor location. All patients underwent regional lymph node dissection. The resectability status was classified according to the NCCN guidelines [8]. The pathological stage was assessed according to the TNM classification system of the Union for International Cancer Control, 8th edition [10]. The preoperative CRP albumin ratio (CAR) was calculated for all cases as a nutritional indicator [11]. The cutoff for the continuous variable was set at the median. RFS was measured from the date of resection to the date of death due to any cause, recurrence, or metastasis. Patients who survived without recurrence were censored. To estimate OS, patients who remained alive were censored on the date of the last follow-up.

### Chemotherapy regimens

Preoperative chemotherapy was not administered routinely until 2014. Subsequently, only patients registered in the Prep-02/JSAP-05 trial [3] received two cycles of gemcitabine and S-1 (GS). As of 2019, preoperative chemotherapy with GS has been implemented as standard practice, following the disclosure of its results. The first-line regimen for borderline resectable pancreatic cancer is mainly gemcitabine and nab-paclitaxel (GA) or fluorouracil and leucovorin and irinotecan and oxaliplatin (FOLFIRINOX) [1]. However, gemcitabine monotherapy is used depending on the patient’s general condition.

The AC regimen used for each patient was S-1 monotherapy. The regimen consisted of S-1 at a dose of 80–120 mg/day, depending on body surface area (BSA): 80 mg/day for BSA < 1.25 m^2^, 100 mg/day for BSA 1.25–1.50 m^2^, and 120 mg/day for BSA > 1.50 m^2^. S-1 was administered twice daily for 4 weeks, followed by a 2-week withdrawal, and repeated every 6 weeks for four cycles. Alternatively, it could be administered twice daily for 2 weeks, followed by a 1-week withdrawal, and repeated every 3 weeks for eight cycles. The AC was scheduled for 24 weeks (total period: 112 days); however, the attending physician had discretion over the actual duration of administration. AC was initiated immediately after the patient’s physical condition improved.

The attending physician determined the dose reduction or discontinuation of AC based on adverse events, following the Common Terminology Criteria for Adverse Events (CTCAEv5.0) [12], recurrence, death from any cause, postoperative performance status (PS), or patient preference. If CTCAE Grade ≥ 3 adverse events occurred, S-1 was withheld until symptoms were resolved or improved. Upon resuming treatment, the S-1 dose was generally reduced by one dose level. For patients with low PS, a dose reduction by one or two levels was considered from the initiation of treatment. If dose reduction below the minimum volume of 80 mg/day was required, S-1 was discontinued. In addition, renal function was considered in determining the volume; creatinine clearance was calculated using the Cockcroft-Gault formula: one dose reduction for ≥ 30 and < 60 mL/min, and two dose reductions for < 30 mL/min.

### Postoperative follow-up

After pancreatectomy, each patient was followed-up at their respective hospital. Tumor markers were evaluated monthly and computed tomography (CT) scans were performed every 6 months during AC, and subsequently every 3 months and 6 months after AC, respectively, until 5 years after surgery. After this period, tumor markers were evaluated and CT scans were performed every 6 months. Recurrence was diagnosed based on findings from CT scans or fludeoxyglucose F18-positron emission tomography/CT. Tumor recurrence was classified as local, distant, or both. Local recurrence was defined as the presence of enlarged soft tissue shadows or lymph nodes around the celiac, hepatic, splenic, or superior mesenteric arteries in the peripancreatic region.

### Statistical analysis

A multinomial logistic model was used to calculate the probability that a patient would have a low or high TDI and to address initial confounding factors. Propensity was estimated by using a multinomial logistic model with the TDI as the dependent variable and the following baseline factors as independent variables: sex, age, presence of diabetes mellitus, preoperative CA19-9 level, CRP albumin ratio (CAR), resectability status (Resectable [R] or Borderline resectable [BR]), with or without AC, tumor location, pathological T-factor, pathological N-factor, histopathological differentiation, residual tumor (R0; resection for cure or complete remission, or R1; microscopic residual tumor), postoperative CA19-9 level, initial S-1 dose, time from surgery to start of AC, reason for AC reduction or discontinuation. Inverse probability of treatment weighting (IPTW) was then applied to balance baseline characteristics between the low TDI and high TDI groups. The balance of covariates between the TDI groups before and after IPTW adjustment was assessed using a standardized difference (SD) approach. An SD of 0.1 indicated negligible differences between weighted estimates of patient characteristics and TDI. IPTW-adjusted Kaplan–Meier curves and log-rank tests were used to compare RFS and OS between patients with low- and high-TDI. In addition, Kaplan–Meier curves and log-rank tests were used to compare the OS between the n- and rl-TDI groups, and rh-, c-, and o-TDI groups. A Cox proportional hazards model was used to perform univariate and multivariate OS analyses. The correlation between categorical variables was evaluated using Pearson’s χ2 test. Statistical significance was defined as p < 0.05. All statistical analyses were performed using EZR (Saitama Medical Center, Jichi Medical University, Saitama, Japan), a graphical user interface for R (The R Foundation for Statistical Computing, Vienna, Austria), EZR is a modified version of the R commander, designed to include statistical functions frequently used in biostatistics.

## Results

### Patient selection and characteristics

**Fig. 1:**
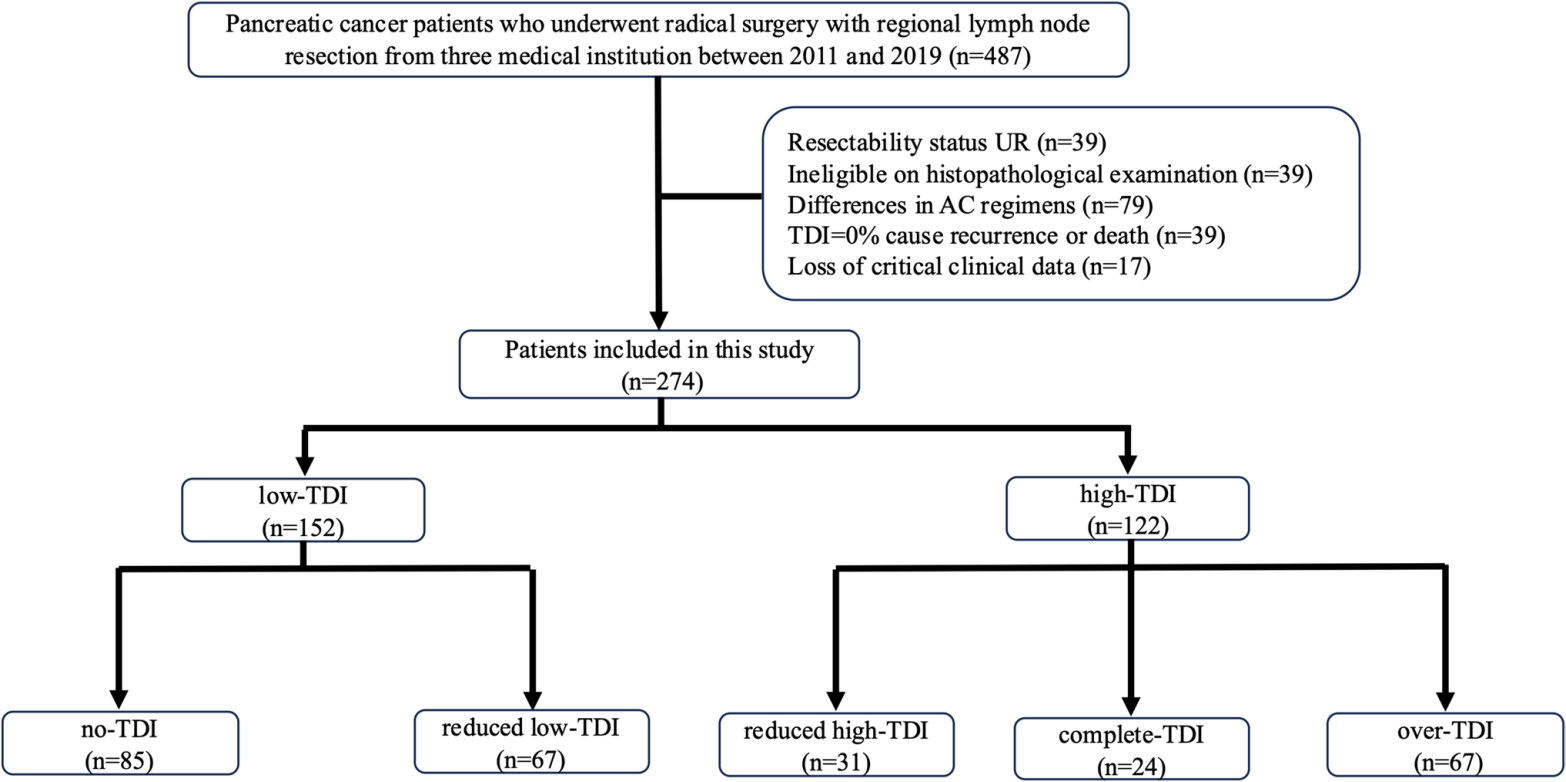
Flow diagram of patient selection and grouping. Of the 487 patients, 213 were excluded based on the exclusion criteria. Of the remaining 274 patients, 152 and 122 were low- and high-TDI, respectively

A total of 487 patients with pancreatic cancer were enrolled in this study, all of whom underwent radical resection, including regional lymph node dissection. After applying exclusion criteria, 213 patients were excluded. Figure 1 illustrates the flow diagram outlining patient selection and categorization. Among the remaining 274 patients, 152 and 122 were classified as having low- and high-TDI, respectively. Patient background in each group is shown in Table 1. After applying IPTW (Table 2), the SD for all covariates was < 0.1, except for the initial dose of S-1 = 80 mg/day, which exhibited an SD = 0.117. The overall balance between the groups was significantly improved, though a slight imbalance persisted.

**Table 1:** Patient characteristics of each TDI

	Low TDI		High TDI		
	n-TDI (N = 85)	rl-TDI (N = 67)	rh-TDI (N = 31)	c-TDI (N = 24)	o-TDI (N = 67)
Preoperative parameter					
Sex, male	45 (53)	40 (60)	12 (39)	11 (48)	37 (55)
Age, ≥ 70 years	69 (81)	40 (60)	21 (68)	13 (56)	31 (46)
DM, yes	30 (35)	17 (25)	11 (35)	6 (26)	21 (31)
Preoperative CA19-9 value, > 120 U/mL	43 (51)	43 (64)	17 (55)	10 (43)	30 (45)
CAR, > 0.085	46(54)	35 (52)	13 (42)	7 (30)	30 (45)
Resectability, BR	22 (26)	14 (21)	6 (19)	4 (17)	11 (16)
Preoperative chemotherapy, yes	9 (11)	9 (13)	5 (16)	1 (4)	2 (3)
Tumor location, head	66 (78)	56 (83)	20 (65)	12 (52)	47 (70)
Histopathological parameter					
Pathological T-factor, 3	37 (44)	22 (33)	7 (22)	9 (39)	24 (36)
Pathological N-factor, ≥ 1	47 (55)	55 (82)	25 (80)	19 (79)	53 (79)
Poorly differentiated adenocarcinoma, yes	3 (4)	16 (24)	2 (6)	2 (9)	7 (10)
Residual tumor, R1	22 (26)	19 (28)	6 (19)	2 (9)	14 (21)
Postoperative parameter					
Postoperative CA19-9 value, > 120 U/mL	–	19 (28)	4 (13)	1 (4)	4 (6)
Initial S-1 dose, = 80 mg/day	–	26 (39)	12 (39)	6 (26)	13 (19)
Time to start AC, ≥ 42 days	–	36 (54)	15 (48)	7 (30)	25 (37)
Reason for S-1 reduction or discontinuation					
PS decline	21 (25)	0 (0)	2 (7)	–	–
Recurrence	–	15 (22)	8 (26)	–	–
Death	–	1 (1)	1 (3)	–	–
Adverse events	–	37 (55)	11 (35)	–	–

*TDI* total dose intensity, *DM* diabetes mellitus, *BR* borderline resectable, *PS* performance status, *AC* adjuvant chmetherapy

**Table 2:** Weighting population

		Unweighted study population			Weighted study population		
		Low TDI (N = 152)	High TDI (N = 122)	SD	Low TDI (N = 142)	High TDI (N = 128)	SD
Preoperative parameter							
Sex, n (%)	Male	85 (56)	60 (49)	0.135	77 (54)	64 (50)	0.081
	Female	67 (44)	62 (51)		65 (46)	64 (50)	
Age, n (%)	≥ 70 y	109 (72)	66 (54)	0.371	95 (67)	83 (65)	0.041
	< 70 y	43 (28)	56 (46)		47 (33)	45 (35)	
DM	Yes	47 (31)	38 (31)	0.005	46 (32)	36 (28)	0.085
	No	107 (69)	84 (69)		96 (68)	92 (72)	
Preoperative CA19-9, n (%)	> 120 U/ml	87 (57)	58 (48)	0.195	76 (54)	73 (57)	0.066
	≤ 120 U/ml	65 (43)	64 (52)		66 (44)	55 (43)	
CAR, n (%)	> 0.85	81 (53)	50 (41)	0.248	71 (50)	68 (53)	0.071
	≤ 0.85	71 (47)	72 (59)		71 (50)	60 (47)	
Resectability, n (%)	BR	36 (24)	22 (18)	0.139	31 (22)	26 (21)	0.031
	R	116 (76)	100 (82)		111 (78)	102 (79)	
Preoperative chemotherapy, n (%)	Yes	18 (12)	9 (7)	0.152	16 (11)	16 (13)	0.047
	No	134 (88)	113 (93)		126 (89)	112 (87)	
Tumor location, n (%)	Head	122 (80)	79 (65)	0.353	108 (76)	97 (77)	0.003
	Body or Tail	30 (20)	43 (35)		34 (24)	31 (23)	
Histopathological parameter							
Pathological T-factor, n (%)	2 ≤	93 (61)	60 (49)	0.091	91 (64)	86 (67)	0.071
	3	59 (39)	62 (51)		51 (36)	42 (33)	
Pathological N-factor, n (%)	0	50 (33)	25 (21)	0.283	39 (27)	35 (27)	0.007
	1 ≥	102 (67)	97 (79)		103 (73)	93 (73)	
Histological differentiation, n (%)	Poorly	19 (12)	11 (9)	0.113	16 (11)	11 (9)	0.091
	Well or moderately	133 (88)	111 (91)		126 (89)	117 (91)	
Residual tumor, n (%)	R0	111 (73)	100 (82)	0.215	106 (75)	101 (79)	0.090
	R1	41 (27)	22 (18)		36 (25)	27 (21)	
Postoperative parameter							
Postoperative CA19-9, n (%)	> 120 U/ml	40 (27)	9 (8)	0.523	27 (19)	22 (17)	0.048
	≤ 120 U/ml	112 (73)	113 (92)		115 (81)	106 (83)	
Initial S-1 dose, n (%)	80 mg/day	26 (17)	31 (25)	0.204	110 (77)	92 (72)	0.117
	> 80 mg/day	126 (83)	91 (75)		32 (23)	36 (28)	
Time to AC, n (%)	≥ 42 days	134 (88)	77 (63)	0.610	113 (79)	99 (78)	0.054
	< 42 days	18 (11)	45 (37)		29 (21)	29 (22)	
S-1 reduction or discontinuation reason							
PS decline, recurrence, death, or adverse events	Yes	74 (48)	22 (17)	0.696	57 (40)	50 (39)	0.015
	No	78 (52)	100 (83)		85 (60)	78 (61)	

*TDI* total dose intensity, *CAR* CRP albumin ratio, *AC* adjuvant chemotherapy, *PS* performance status

**Fig. 2:**
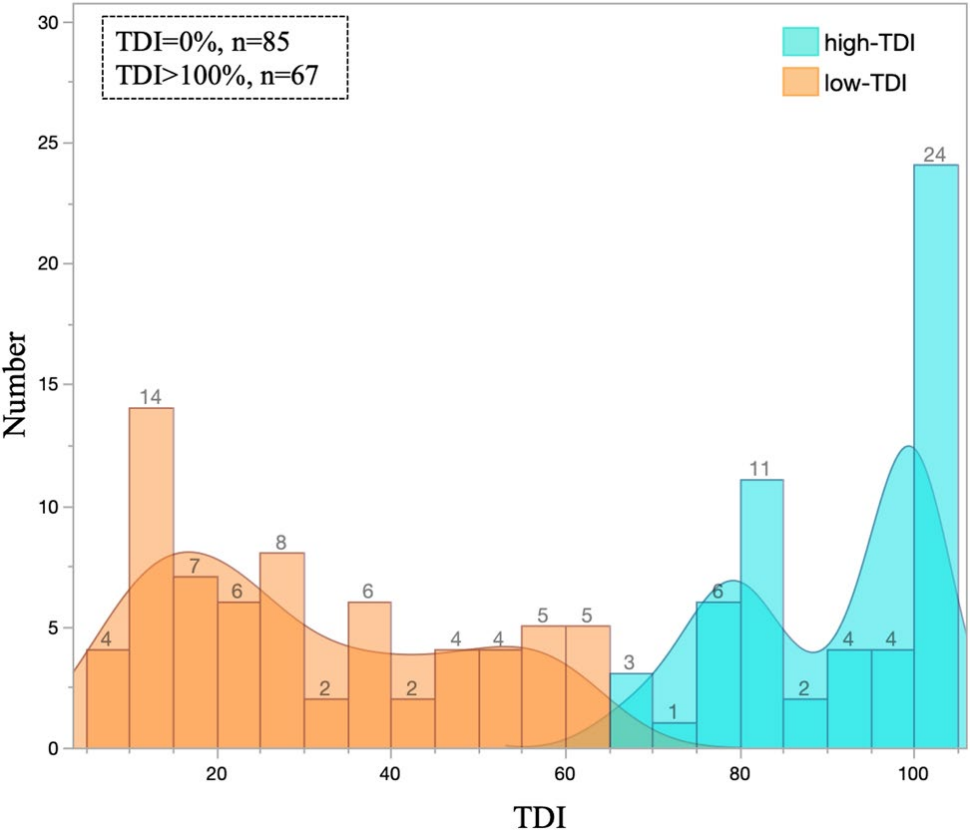
Histogram and Kernel density estimates by TDI. High-TDI is shown in blue and low-TDI is shown in orange. Kernel density estimates showed that high-TDI was bimodal at 80–85% (9% of total) and 100% (20% of total), while low-TDI was unimodal at 10–20% (14% of total)

A histogram of the TDI and Kernel density estimates is shown in Fig. 2. The Kernel density estimates exhibited a bimodal distribution for rh-TDI at 80–85% and 100%, whereas rl-TDI displayed a unimodal distribution at 10–20%.

### Reasons for incomplete adjuvant chemotherapy

In total, 31 (25%) and 152 (100%) patients in the high- and low-TDI groups, respectively, did not complete AC. Table 3 summarizes the reasons for discontinuation. The primary reasons for cessation of AC were tumor recurrence (high TDI: 8 patients, 26%; low TDI: 15 patients, 10%; p = 0.003), adverse events (high TDI: 11 patients, 35%; low TDI: 37 patients, 24%; p = 0.262), and low performance status (high TDI: 2 patients, 7%; low TDI: 21 patients, 14%; p = 0.377).

**Table 3:** Causes of failure to complete adjuvant chemotherapy

	Low TDI (N = 152)	High TDI (N = 31)	P value
Recurrence	15 (10)	8 (26)	0.003^†^
Adverse event (CTCAE Grade 3 or 4)	37 (24)	11 (35)	0.262
Nausea	3 (8)	2 (18)	
Diarrhea	15 (41)	2 (18)	
Pancytopenia	2 (6)	1 10)	
Stomatitis, Tastophagia	7 (19)	3 (27)	
Dysgeusia	1 (3)	3 (27)	
Fatigue	5 (13)	0 (0)	
Peripheral neuropathy	2 (5)	0 (0)	
Drug-induced interstitial pneumonia	2 (5)	0 (0)	
Age	13 (9)	0 (0)	0.132
Cholangitis	5 (3)	1 (3)	1.000
Renal disfunction	8 (5)	1 (3)	1.000
Postoperative pancreatogenic diabetes mellitus	3 (2)	1 (3)	0.527
Low performance status	21 (14)	2 (7)	0.377
Patient decision to discontinue treatment	24 (15)	2 (7)	0.260
Death	1 (1)	1 (3)	0.311
Others*	25 (17)	4 (13)	0.790

*TDI* total dose intensity, *CTCAE*, Common Terminology Criteria for Adverse Events

^*^Development of other diseases unrelated to chemotherapy, or prolonged hospitalization due to postoperative complications

^†^Statistically significant

### Survival outcomes and post-relapse treatment outcomes

**Fig. 3:**
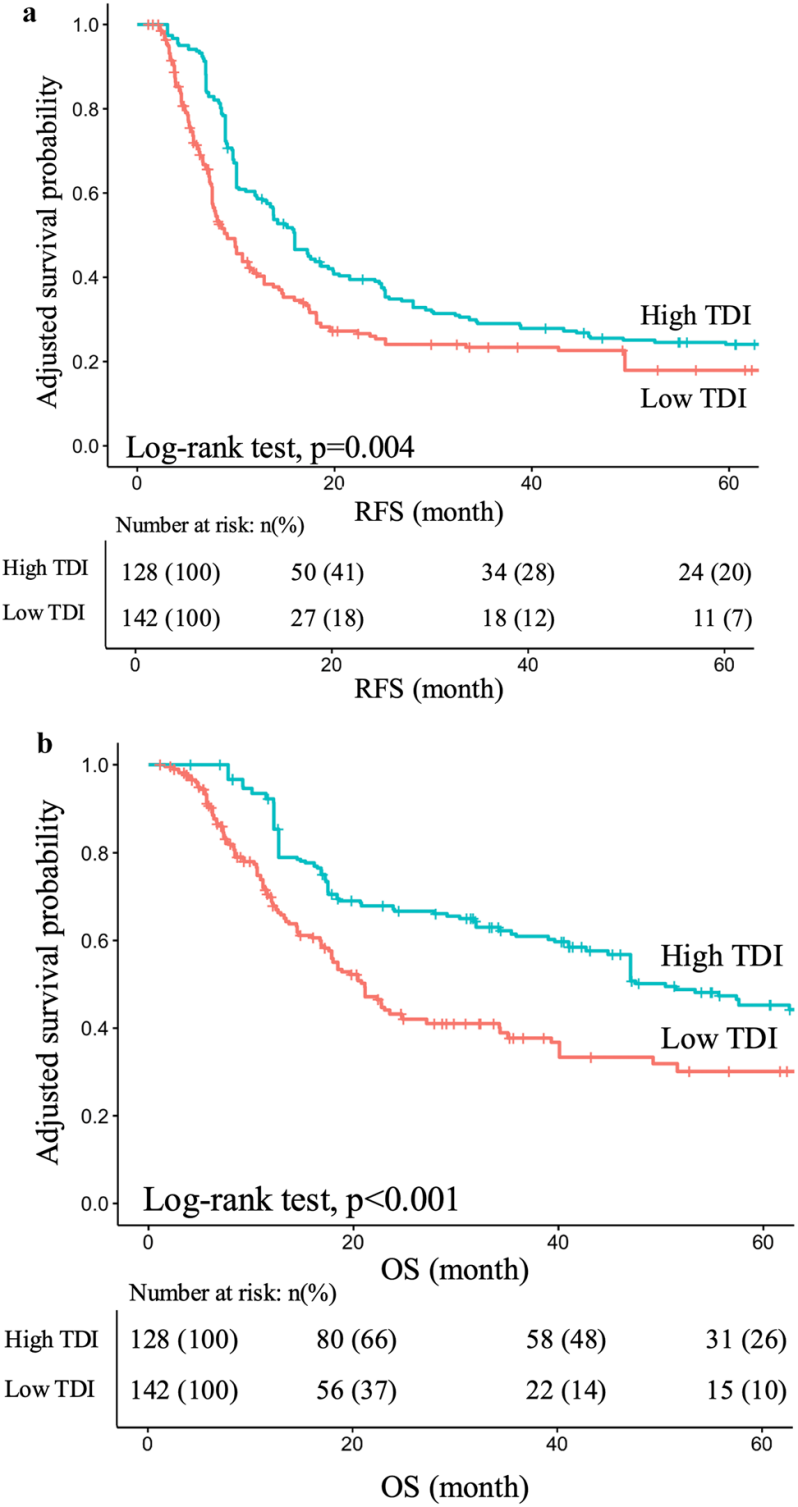
**a** IPTW-adjusted RFS. The high-TDI group showed significantly better RFS than the low-TDI group (high-TDI: median RFS, 18 months; low-TDI: median RFS, 8 months; p = 0.004). **b** IPTW-adjusted OS. The high-TDI group showed significantly better OS than the low-TDI group (high-TDI: MST = 50 months; low-TDI: MST = 20 months; p < 0.001)

The RFS and OS of the low- and high-TDI groups are shown in Fig. 3. The median RFS in the low- and high-TDI groups was 8 and 18 months, respectively (p = 0.004) (Fig. 3a). The median survival times (MST) for the low- and high-TDI groups were 20 and 50 months, respectively (p < 0.001) (Fig. 3b).

**Fig. 4:**
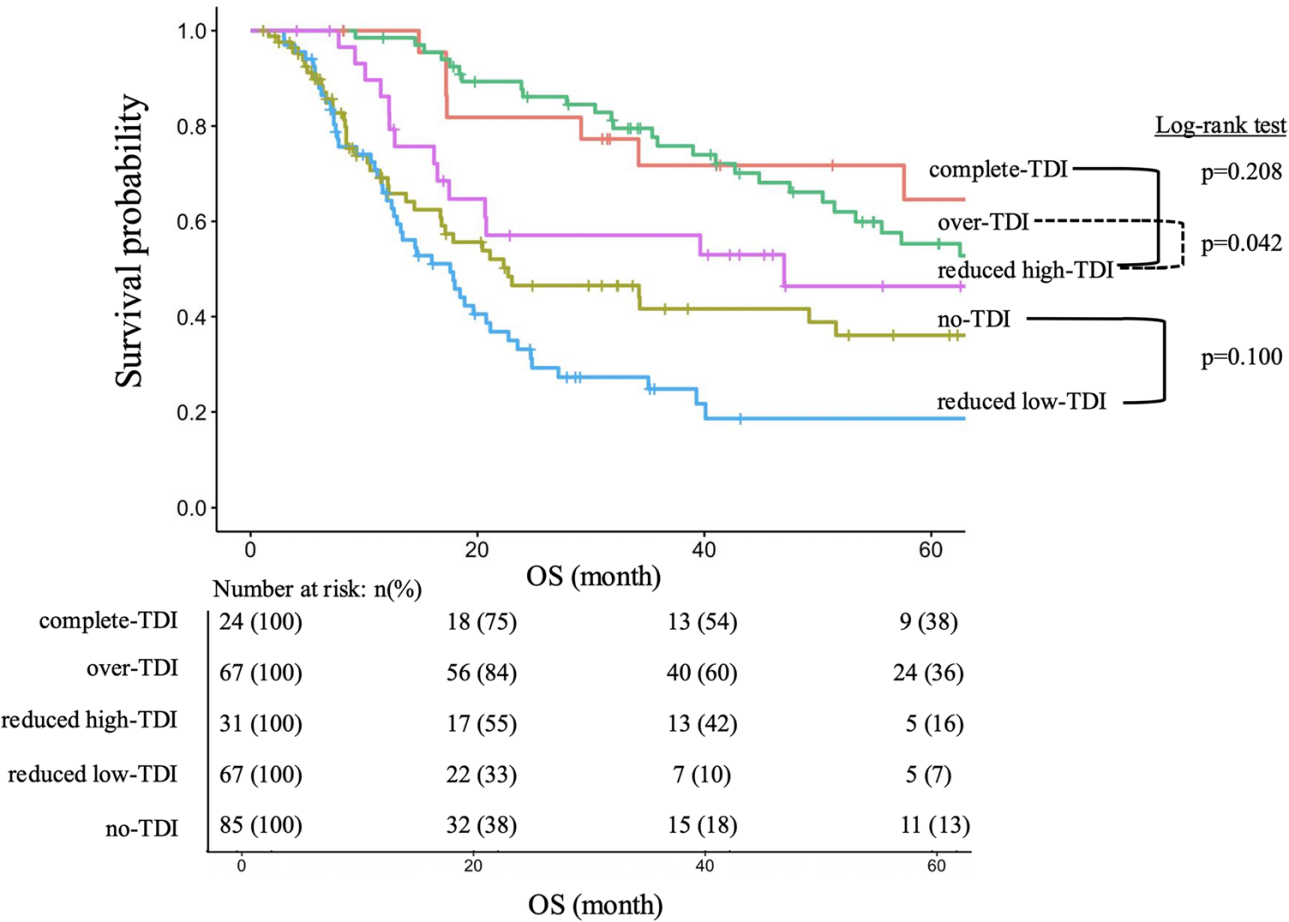
Overall survival (OS) of patients according to the TDI of adjuvant chemotherapy (AC). There were no significant differences between the reduced high- and complete-TDI groups (reduced high TDI: median survival time (MST) = 47 months; complete TDI: MST = 72 months; p = 0.208). There were significant differences between the no- and reduced low-TDI groups (no TDI: MST = 21 months; reduced low TDI: MST = 18 months; p = 0.010)

The MST for the n-, rl-, rh-, c- and o-TDI groups were 21, 18, 47, 72 and 61 months, respectively (n- vs rl-TDI, p = 0.010; rh- vs c-TDI, p = 0.208; rh- vs o-TDI, p = 0.042) (Fig. 4). The presence and location of recurrence as well as post-recurrence treatments for the each TDI groups, are shown in Table 4. There were no substantial differences in recurrence location between low- and high-TDI group. However, more patients in high-TDI group received treatment after recurrence compared with low-TDI group (p < 0.001). In the comparison between n- and rl-TDI, recurrence was significantly more frequent in the latter (p < 0.001), and the proportion of patients who received treatment for recurrence was higher in the latter group (p = 0.045).

**Table 4:** Recurrence status and treatment after relapse

	Low TDI		High TDI			Low- vs. High-TDI P value	n- vs. rl-TDI P value	rh- vs. c-TDI P value
	n-TDI (N = 85)	rl-TDI (N = 67)	rh-TDI (N = 31)	c-TDI (N = 24)	o-TDI (N = 67)			
Recurrence, n (%)	46 (54)	58 (88)	21 (68)	16 (70)	50 (75)	0.692	< 0.001^†^	1.000
Recurrence free deaths, n (%)	15 (18)	4 (6)	2 (6)	1 (4)	1 (1)	0.007^†^	0.046^†^	1.000
Recurrence location, n (%)								
Local and Distant recurrence	4 (8)	5 (9)	5 (24)	1 (6)	3 (6)	0.805	1.000	0.206
Local recurrence	17 (37)	20 (34)	5 (24)	7 (44)	20 (40)	0.881	0.838	0.151
Distant recurrence	25 (55)	33 (57)	11 (52)	8 (50)	27 (56)	0.771	0.844	0.939
Sites of distant recurrence, n (%)								
Liver	18 (39)	19 (33)	6 (29)	4 (25)	11 (22)	0.114	0.541	0.899
Lung	6 (13)	9 (16)	7 (33)	3 (19)	12 (24)	0.067	0.273	0.612
Para-aortic lymph node	0 (0)	0 (0)	1 (5)	0 (0)	0 (0)	0.455	1.000	0.679
Peritoneal dissemination	6 (13)	11 (19)	3 (14)	1 (6)	8 (12)	0.689	0.594	0.696
Other	1 (2)	2 (3)	2 (10)	0 (0)	1 (2)	1.000	1.000	0.551
Treatment after recurrence, n (%)	14 (30)	30 (52)	15 (71)	14 (88)	42 (84)	< 0.001^†^	0.045^†^	0.239
Surgery	0 (0)	2 (7)	0 (0)	3 (21)	9 (21)	0.076	1.000	0.058
Systemic chemotherapy	10 (71)	24 (80)	14 (93)	10 (71)	32 (76)	0.130	0.701	0.118
HAIC	4 (29)	4 (13)	1 (7)	1 (8)	1 (2)	0.406	0.242	0.959

*TDI* total dose intensity, *HAIC* hepatic arterial infusion chemotherapy

^†^Statistically significant

There were no substantial differences in recurrence location, or the frequency in the treatment after recurrence between rh-TDI and c-TDI groups.

### Univariate and multivariate analyses for the predictors of poor OS

Univariate and multivariate analyses of the predictors of poor OS are shown in Table 5. Univariate analysis revealed that preoperative CA19-9 value > 120 U/mL, CAR > 0.085, BR status, preoperative chemotherapy, pancreatic head cancer, pathological T3, lymph node metastases, poorly differentiated adenocarcinoma, postoperative CA19-9 value > 120 U/mL, initial dose of S-1 of 80 mg/day, low TDI, and recurrence were associated with poor OS. Multivariate analysis revealed that lymph node metastases (HR = 1.83; 95%CI, 1.408–5.697; p = 0.003), postoperative CA19-9 value > 120 U/mL (HR = 3.00; 95%CI, 1.768–5.108; p < 0.001), low TDI (HR = 2.94; 95%CI, 1.856–4.657; p = 0.001), and recurrence (HR = 2.83; 95%CI, 1.422–5.636; p = 0.003) were independent predictors of poor OS.

**Table 5:** Univariate and multivariate analyses of OS

Parameter	Univariate	Multivariate	
	P value	HR (95% CI)	P value
Sex, male	0.202		
Age, ≥ 70 years	0.771		
DM, yes	0.846		
Preoperative CA19-9 value, > 120 U/mL	0.010^†^	1.16 (0.761–1.767)	0.489
CAR, > 0.085	0.076	1.21 (0.797–1.846)	0.366
Resectability, BR	0.001^†^	1.59 (0.936–2.715)	0.085
Preoperative chemotherapy, yes	0.008^†^	1.29 (0.617–2.708)	0.495
Tumor location, head	< 0.001^†^	1.49 (0.855–2.616)	0.157
Pathological T-factor, 3	0.068	1.15 (0.727–1.832)	0.540
Pathological N-factor, ≥ 1	< 0.001^†^	1.83 (1.408–5.697)	0.003^†^
Histological differentiation, poorly	0.006^†^	1.56 (0.892–2.732)	0.118
Residual tumor, R1	0.327		
Postoperative CA19-9 value, > 120 U/mL	< 0.001^†^	3.00 (1.768–5.108)	< 0.001^†^
Initial dose of S-1, ≤ 80 mg/day	0.029^†^	1.44 (0.925–2.272)	0.105
Time to start AC, ≥ 42 days	0.913		
TDI, < 62.5%	< 0.001^†^	2.94 (1.856–4.657)	< 0.001^†^
Recurrence, yes	< 0.001^†^	2.83 (1.422–5.636)	0.003^†^

*OS* overall survival, HR hazard ratio, *CI* conflict of interest, *DM* diabetes mellitus, *CAR* CRP albumin ratio, *BR* borderline resection, *AC* adjuvant chemotherapy, *TDI* total dose intensity

^†^Statistically significant

### Univariate and multivariate analyses for the predictors of low TDI

Univariate and multivariate analyses were conducted to identify predictors of low TDI (Supplemental Table 1). The univariate analysis revealed that age ≥ 70 years, CAR > 0.085, pancreatic head cancer, lymph node metastases, postoperative CA19-9 levels > 120 U/mL, S-1 dose reduction or discontinuation due to decline of PS, and S-1 dose reduction or discontinuation due to adverse events were associated with low TDI. Multivariate analysis identified pancreatic head cancer (HR, 4.86; 95% CI, 1.697–13.92; p = 0.003), postoperative CA19-9 levels > 120 U/mL (HR, 4.47; 95% CI, 1.655–12.07; p = 0.003), and S-1 discontinuation due to adverse events (HR, 13.3; 95% CI, 5.470–32.71; p < 0.001) as independent risk factors for low TDI.

## Discussion

In this study, we demonstrated that a TDI of ≥ 62.5% was associated with improved RFS and OS across multicenter, weighted patient cohorts analyzed using IPTW. Prognosis did not substantially differ between the reduced-dose group (TDI ≥ 62.5%) and those with TDI = 100%. In addition, multivariate analysis revealed that low TDI is an independent predictor of poor OS. These results suggest that a moderate dose reduction may still yield outcomes comparable to those of patients completing full-dose adjuvant chemotherapy.

The standard postoperative adjuvant chemotherapy in Japan is S-1; however, few studies have evaluated the impact of dosage on prognosis [13–16]. Relative dose intensity (RDI) is commonly used to measure AC dosage for pancreatic cancer [17], with an RDI of 80% reportedly associated with a good prognosis [15]. However, the optimal dosage and dosage duration remain unclear. Our previous study suggested that a high TDI was associated with a significantly better OS [7]. However, a low TDI may be utilized in cases where S-1 was discontinued due to recurrence, which has been associated with an MST of 9.3 months [18]; potentially contributing to the poor prognosis for patients in this group. In this cohort, the risk factors for low TDI, based on multivariate analysis, were pancreatic head cancer (p = 0.003), postoperative CA19-9 levels (p = 0.003), and adverse events associated with S-1 (p < 0.001). Although the low-TDI group is expected to have included a higher number of cases with patient backgrounds associated with poor prognostic factors, IPTW was employed to minimize the impact of background differences between the groups. In the present IPTW adjustment, the overall balance between the groups was substantially improved; however, the initial dose of S-1 = 80 mg/day still exhibited SD > 0.1. Nevertheless, multivariable analysis did not identify the initial dose of S-1 as an independent predictor of poor OS (p = 0.105). Given this, although complete homogeneity between the groups might have not been achieved, this residual imbalance is unlikely to have a significant impact on the OS assessment.

Our previous study did not mention the distribution of TDI among patients with high TDI [7], and a better prognosis was influenced by a substantial proportion of patients with TDI = 100%. In this study, the most common category of high TDI was TDI > 100% (55%), followed by TDI = 100% (22%). We examined OS for patients with TDI = 100% (c-TDI) and 62.5% ≤ TDI < 100% (rh-TDI), finding no significant difference between the two groups (p = 0.208). In Stage III colorectal cancer, the ACHIEVE trial [19] suggested that shorter durations of AC were not poor prognostic factors for patients at low risk of recurrence. The results of our study also suggest that a reduced AC dose may be possible for pancreatic cancer. However, in the present study, a significant difference in OS was observed between the rh-TDI and o-TDI groups (p = 0.042). Although there appears to be difference in the patient backgrounds between the two groups, there is room for debate regarding whether 62.5% of the ideal dose can be considered the optimal dose for improving OS. Tomimaru et al. reported no difference in prognosis between 6 months of postoperative adjuvant S-1 chemotherapy (TDI = 100%) and treatment extending beyond 6 months (TDI > 100%) [20]. Based on the findings and the results of this study, it was suggested that the prognostic effect of S-1 as adjuvant chemotherapy may be limited beyond a certain dosage threshold. Future large-scale prospective studies are required to determine the optimal minimum effective dose.

Univariate and multivariate analyses identified the pathological N-factor, postoperative CA19-9 value, low TDI, and recurrence as independent predictors of poor OS. Previous reports indicate that the pathological N-factor correlates with the mode and timing of postoperative recurrence of pancreatic cancer [21, 22]. Postoperative elevated CA19-9 levels are also considered to be a potential factor suggesting recurrence [23]. This suggests that, as expected, recurrence is an important factor contributing to poor prognosis. Kim et al. reported that treatment after recurrence is an important factor affecting OS [24]. In this study, no significant difference was found in the site of recurrence; however, post-relapse treatment was less frequent in the low TDI group compared to the high TDI group (29% vs. 58%, p < 0.001). The efficacy of reoperation, systemic chemotherapy, radiotherapy, and chemoradiotherapy in the treatment of postoperative recurrence has been reported [25–28]. Post-relapse therapy positively impacts OS [29] and is a predictor of favorable outcomes. One reason for the higher post-recurrence treatment rate in the high TDI group could be the longer RFS observed in this group. The low TDI group included more cases of early recurrence, defined as occurring within 12 months of surgery [30, 31]. Patients experiencing early relapse received consecutive treatment with AC, followed by more intensive post-relapse therapy. Consequently, these patients may have had lower tolerance, which may have hindered their ability to receive effective post-relapse therapy. Previous studies have reported that perioperative malnutrition is a poor prognostic factor [32] and can lead to failure in completing AC [33]. In this study, CAR exhibited a trend toward being a poor prognostic factor in univariate analysis (p = 0.076). Optimizing perioperative nutritional management to maintain a high TDI may help reduce early recurrence and improve prognosis. Furthermore, with TDI = 62.5% as a reference, dose reduction or discontinuation should be considered without hesitation in the presence of S-1-related adverse events. Enhancing the patient’s general condition for post-recurrence treatment may further contribute to improved prognosis.

This study has certain limitations. First, although this was a multicenter study, it was retrospective in nature. Although IPTW was employed to address differences in patient backgrounds between low- and high-TDI groups, potential bias remains due to unaccounted factors. Second, the sample sizes in the rh-TDI and c-TDI groups were small. In the present study, there was no difference in OS between the rh- and c-TDI groups. However, the reproducibility of this finding with a larger patient population remains uncertain.

## Conclusion

In this study, we investigated the prognostic impact of adjuvant S-1 chemotherapy dosage following surgery for pancreatic cancer across multiple centers, employing IPTW to account for patient differences. A TDI of ≥ 62.5% emerged as a crucial criterion for favorable prognosis. It was suggested that the prognostic benefit of adjuvant chemotherapy may be limited beyond a certain dosage threshold.

## Data Availability

The datasets used and/or analyzed during the current study are available from the corresponding author upon reasonable request.
